# Segmentation Errors in the Measurement of Volumetric Parameters by Swept-Source Anterior Segment Optical Coherence Tomography

**DOI:** 10.3389/fmed.2021.761550

**Published:** 2021-12-17

**Authors:** Hailiu Chen, Jie Meng, Peng Lu, Dan Ye, Yunxuan Li, Lu Cheng, Yangyunhui Li, Xiaoling Liang, Wenyong Huang, Jingjing Huang

**Affiliations:** ^1^State Key Laboratory of Ophthalmology, Zhongshan Ophthalmic Center, Sun Yat-sen University, Guangdong Provincial Key Laboratory of Ophthalmology and Visual Science, Guangdong Provincial Clinical Research Center for Ocular Diseases, Guangzhou, China; ^2^Department of Ophthalmology, Joint Shantou International Eye Center, Shantou University, Chinese University of Hong Kong, Shantou, China

**Keywords:** segmentation error, anterior chamber volume, iris volume, swept-source anterior segment optical coherence tomography, narrow angle

## Abstract

**Purpose:** To investigate the error rate of segmentation in the automatic measurement of anterior chamber volume (ACV) and iris volume (IV) by swept-source anterior segment optical coherence tomography (SS-ASOCT) in narrow-angle and wide-angle eyes.

**Methods:** In this study, fifty eyes from 25 narrow-angle subjects and fifty eyes from 25 wide-angle subjects were enrolled. SS-ASOCT examinations were performed and each SS-ASOCT scan was reviewed, and segmentation errors in the automatic measurement of ACV and IV were classified and manually corrected. Error rates were compared between the narrow-angle and the wide-angle groups, and ACV and IV before and after manual correction were compared.

**Results:** A total of 12,800 SS-ASOCT scans were reviewed. Segmentation error rates of angle recess, iris root, posterior surface of the iris, pupil margin, and anterior surface of the lens were 84.06, 93.30, 13.15, 59.21, and 25.27%, respectively. Segmentation errors of angle recess, iris root, posterior surface of the iris, and pupil margin occurred more frequently in narrow-angle eyes, while more segmentation errors of the anterior surface of the lens were found in wide-angle eyes (all *P* < 0.001). ACV decreased and IV increased significantly after manual correction of segmentation errors in both groups (all *P* < 0.01).

**Conclusion:** Segmentation errors were prevalent in the volumetric measurement by SS-ASOCT, particularly in narrow-angle eyes, leading to mismeasurement of ACV and IV.

## Introduction

Anterior segment optical coherence tomography (AS-OCT) is a non-contact, rapid imaging device that uses low-coherence interferometry to obtain cross-sectional images of the anterior segment (1). The swept-source anterior segment OCT (SS-ASOCT) delivers high-resolution images of the anterior segment along a large image depth, at a fast acquisition speed (2). The high scan speed facilitates 360 degrees imaging of the anterior segment, providing a more precise and representative measurement of the anterior chamber volume (ACV) and iris volume (IV) (3).

Recent studies have found that ACV and IV were important parameters in the screening, diagnosis, and treatment decisions for narrow-angle or angle-closure patients. Wang et al. reported that the ethnic Chinese tended to have smaller ACV than Caucasians, which was the main contributor to the narrower drainage angle in the Chinese (4). Foo et al. reported that angle width was largely dependent on variation in ACV, anterior chamber area, and lens vault (5). Li et al. tested the power of volumetric parameters to differentiate narrow angle from open angle with gonioscopy as reference standard and found that the patients with narrow angle could be more easily detected using the measurement of ACV (6). These studies indicated that the measurement of ACV could be an important factor in the screening and early detection for anatomically narrow angle, which is beneficial in preventing the development of angle closure glaucoma. Esfandiari et al. reported that eyes with a shallower anterior chamber and thinner irises were more likely to experience angle opening from a laser peripheral iridotomy (LPI), which aided clinicians to decide whether an LPI should be attempted or a primary lens extraction might be indicated for primary angle closure suspects (7). These findings indicated that an accurate and stable measurement of ACV and IV plays an important role in the decision-making of treatment among the narrow-angle subjects.

The built-in segmentation algorithm of SS-ASOCT was commonly used for the automatic measurement of ACV and IV, which detected the anterior and posterior boundaries of the iris and cornea in the individual scans. Previous studies reported that using the automated OCT segmentation algorithm could lead to segmentation errors in the retinal thickness measurement, resulting in the misinterpretation of glaucoma or retinopathy (8, 9). Similarly, in clinical practice, we have noticed quite a few segmentation errors in the automatic measurement of ACV and IV using the built-in caliper software of SS-ASOCT. The inaccuracy of ACV and IV would generate errors in the evaluation of the anatomic characteristics of the angle and iris, leading to unreliable results in both clinical research and the diagnosis or management of subjects with narrow or closed angle. Therefore, in the measurement of ACV and IV, manual adjustment of errors was usually made if the software failed to detect the iris and corneal boundaries at the correct location (3). Moreover, a previous study reported that segmentation errors of OCT were more frequently noticed when the structure was distorted and indistinguishable (10). Analogously, in eyes with narrow angle, the congestion of the anterior segment structure might make it more difficult for the automatic algorithm to identify the border of the anterior segment structure, leading to more segmentation errors.

However, there was no investigation of the prevalence, associated factors, and impact of segmentation errors in volume measurements by SS-ASOCT. In addition, the distribution of segmentation errors in narrow-angle and wide-angle eyes remains unknown. The purpose of the current study was to investigate the error rate of segmentation in the automatic measurement of ACV and IV by SS-ASOCT between narrow-angle and wide-angle eyes, as well as the determinants and the impact of segmentation errors on volume measurements. The study would be helpful to prevent misestimation of the volume parameters determined by SS-ASOCT.

## Methods

### Participants

Participants from the Guangzhou Diabetic Eye Study were included in this cross-sectional study. Details of the protocol and eligibility of Guangzhou Diabetic Eye Study have been described previously (11, 12). Subjects who underwent SS-ASOCT imaging at the Zhongshan Ophthalmic Center of Sun Yat-sen University (Guangzhou, China) between December 2017 and July 2018 were considered in the current analysis. The study protocol was approved by the Institutional Review Board and all procedures conformed to the tenets of the Declaration of Helsinki. Written informed consent was obtained from all participants in the study. All participants underwent complete ophthalmic evaluation, such as best-corrected visual acuity measurement, slit-lamp examination, gonioscopy examination, stereoscopic optic disc examination and fundus evaluation with a 90-diopter lens, fundus photographs by a retinal camera (CR-2; Canon, Tokyo, Japan), and intraocular pressure (IOP) measurement by Goldmann applanation tonometry.

Inclusion criteria for the subjects in the current study were: (1) age between 40 and 80 years; (2) best-corrected visual acuity better than 20/200; (3) spherical equivalent < −6 D, astigmatism <3 D; (4) IOP <21 mmHg; (5) without iridotrabecular contact as verified by gonioscopy and SS-ASOCT; and (6) normal appearance of optic nerve under stereoscopic examination.

Exclusion criteria were: (1) history of ocular surgeries; (2) history of ocular trauma; (3) disorders of anterior segment, such as corneal opacity, iridocyclitis, and lens dislocation; (4) fundus diseases except for diabetic retinopathy; (5) inability to fixate for eye examination; and (6) other systemic diseases except for diabetes mellitus.

Gonioscopy was performed in each subject by two independent glaucoma specialists (JH and WH) using a Goldmann-style one-mirror lens (Model 902; Haag Streit, Bern, Switzerland) with low ambient illumination. The angle width of each eye was classified according to the Shaffer grading system. A narrow angle was defined as Shaffer grade one or lower, and a wide angle was defined as Shaffer grade two or higher in all quadrants (13). If a discrepancy of the classification existed, then, two glaucoma specialists performed a second examination and confirmed the state of the anterior chamber angle. Twenty-five wide-angle and 25 narrow-angle subjects who met the inclusion criteria were included in the current study.

### Swept-Source Anterior Segment Optical Coherence Tomography (SS-ASOCT)

Swept-source anterior segment OCT (CASIA SS-1000 OCT; Tomey, Nagoya, Japan) examinations and measurements were performed by the same trained physician (JM) who was masked to the clinical data. Miotic or mydriatic medications were not used in any of the subjects for at least 7 days prior to imaging. All SS-ASOCT images were taken under dark conditions (0.16 lux measured with a digital luminometer IM-2D; Topcon, Tokyo, Japan) with sitting posture. To avoid eyelid artifact, the operator gently opened the upper and lower lid without compressing the bulb. The participants were asked to fixate on an internal fixation target during the scan, with refractive correction to perform the measurements in an unaccommodated state. Horizontal standard anterior segment single-scan mode (0–180 degrees) was used for perpendicular scans centered on the pupil and was repeated three times. A volume scan comprising of 128 radial scans was used to image the iris and anterior chamber. Alignment that results in a central corneal reflex ensures good repeatability as recommended by the manufacturer. Images with poor quality due to eye movement or lid overlapping were excluded from analysis.

**Figure 1:**
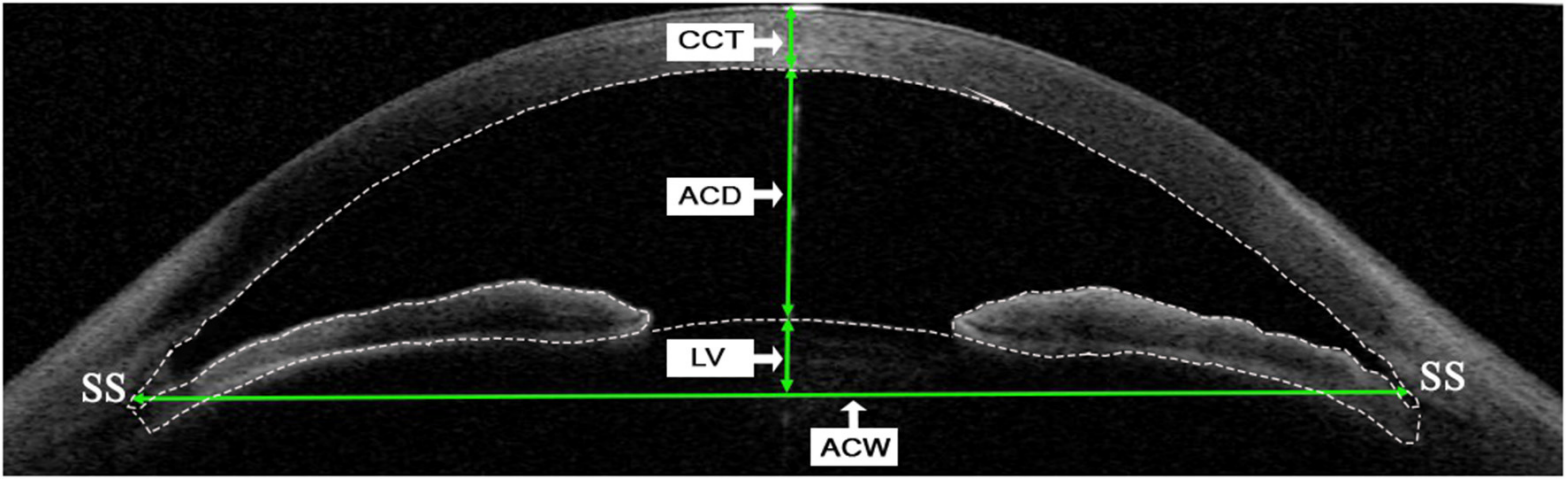
Determinations of linear parameters of the anterior segment by swept-source anterior segment optical coherence tomography (SS-ASOCT). Central corneal thickness (CCT) was defined as a distance from anterior to posterior cornea along a perpendicular line that extends from the median point of the line connecting the two scleral spurs. Anterior chamber depth (ACD) was defined as the axial distance from the corneal endothelium to the anterior lens surface. Lens vault (LV) was defined as the perpendicular distance between the anterior pole of the crystalline lens and the horizontal line connecting the two scleral spurs. Anterior chamber width (ACW) was defined as the distance between the two scleral spurs. The dotted line indicates boundaries of the cornea, iris, and anterior surface of lens, which are detected automatically by the built-in software when measuring the anterior chamber volume (ACV) and iris volume (IV). SS, scleral spur.

Swept-source anterior segment OCT linear parameters were measured at the horizontal (0–180 degrees) and vertical B-scan (90–270 degrees) once the scleral spur was manually marked using the built-in caliper software (V.7J.8; Tomey, Nagoya, Japan) by 1 experienced physician (JM). Briefly, anterior chamber depth (ACD) was defined as the axial distance from the corneal endothelium to the anterior lens surface. Anterior chamber width (ACW) was the distance between the two scleral spurs. Lens vault (LV) was defined as the perpendicular distance between the anterior pole of the crystalline lens and the horizontal line connecting the two scleral spurs. Central corneal thickness (CCT) was defined as a distance from the anterior to posterior cornea along a perpendicular line that extends from the median point of the line connecting the two scleral spurs. Each parameter was measured three times and the average value was recorded. All parameters were calculated by the average of the measurements from the horizontal and vertical cross-sections (Figure 1).

The measurement of IV and ACV was conducted automatically by the instrument software, with 128 radial scans of each eye included in analysis (images were analyzed for every 1.4 degrees). In the measurement process, the instrument software (V.7J.8; Tomey, Nagoya, Japan) automatically detected the anterior and posterior boundaries of the cornea and iris, and the anterior boundary of lens in the individual scan (Figure 1). The iris root was defined as the intersection of the anterior and posterior iris boundaries and the ciliary body. The anterior iris boundary was defined as the anterior chamber-anterior iris surface interface, whereas the posterior iris boundary was defined as the external border of the iris pigment epithelium. The iris and anterior chamber volume were calculated as a summation of pixel volume derived from individual scans by the algorithm of the software (14).

An experienced glaucoma specialist (HC) reviewed each SS-ASOCT scan and manually corrected segmentation errors using the built-in software when the boundaries of cornea, iris, and lens delineated automatically did not conform with the actual borders. Each scan after manual correction was checked by a second experienced glaucoma specialist (JH) to ensure the accuracy of the manual correction. The categories of segmentation errors and the number of scans with each type of segmentation error were recorded in both the narrow-angle and the wide-angle groups. Furthermore, the IV and ACV of each eye were remeasured and recorded after correction of errors.

### Statistical Analysis

Statistical analyses were performed using SPSS software version 25.0 (SPSS, Inc., Chicago, IL, USA). Age between the two groups was compared using an independent *t-*test. Gender and segmentation error rates of all categories between the two groups were compared using the chi-square test. IV and ACV before and after manual correction were compared using the paired *t*-test. Differences before and after manual correction of the actual value of IV and ACV were compared using the chi-square test. In addition, univariate logistic regression analyses were performed. The dependent variables were the segmentation error rates of angle recess, iris root, posterior surface of the iris, pupil margin, and anterior surface of lens, respectively, and the independent variables were SS-ASOCT linear parameters, such as CCT, ACD, ACW, and LV. A *P* < 0.05 was considered statistically significant.

## Results

Fifty eyes from 25 narrow-angle subjects and fifty eyes from 25 wide-angle subjects met the inclusion criteria and were included in the current study. Demographic and biometric characteristics are summarized in Table 1. There were no significant differences concerning age, gender, and CCT between the groups (*P* = 0.653, 0.891, and 0.691, respectively). ACD and ACW were significantly smaller while LV was larger in the narrow-angle group when compared with the wide-angle group (all *P* < 0.001).

**Table 1:** Demographics and clinical characteristics of narrow angle and wide angle eyes.

**Variables**	**Total**	**Narrow-angle**	**Wide-angle**	***P* value**
	**(*N* = 50)**	**(*N* = 25)**	**(*N* = 25)**	
Age (y)	68.6 ± 9.1	68.9 ± 8.0	68.3 ± 9.9	0.653
Gender (male/female)	37/13	18/7	19/6	0.891
ACD (mm)	2.49 ± 0.45	2.08 ± 0.17	2.80 ± 0.33	**<0.001**
CCT (μm)	537 ± 29	536 ± 25	538 ± 31	0.691
LV (mm)	0.60 ± 0.36	0.83 ± 0.20	0.42 ± 0.34	**<0.001**
ACW (mm)	11.88 ± 0.49	11.55 ± 0.38	12.14 ± 0.40	**<0.001**

*Data are presented as mean ± SD. Bold values indicate statistical significance. ACD, anterior chamber depth; CCT, central corneal thickness; LV, lens vault; ACW, anterior chamber width*.

**Figure 2:**
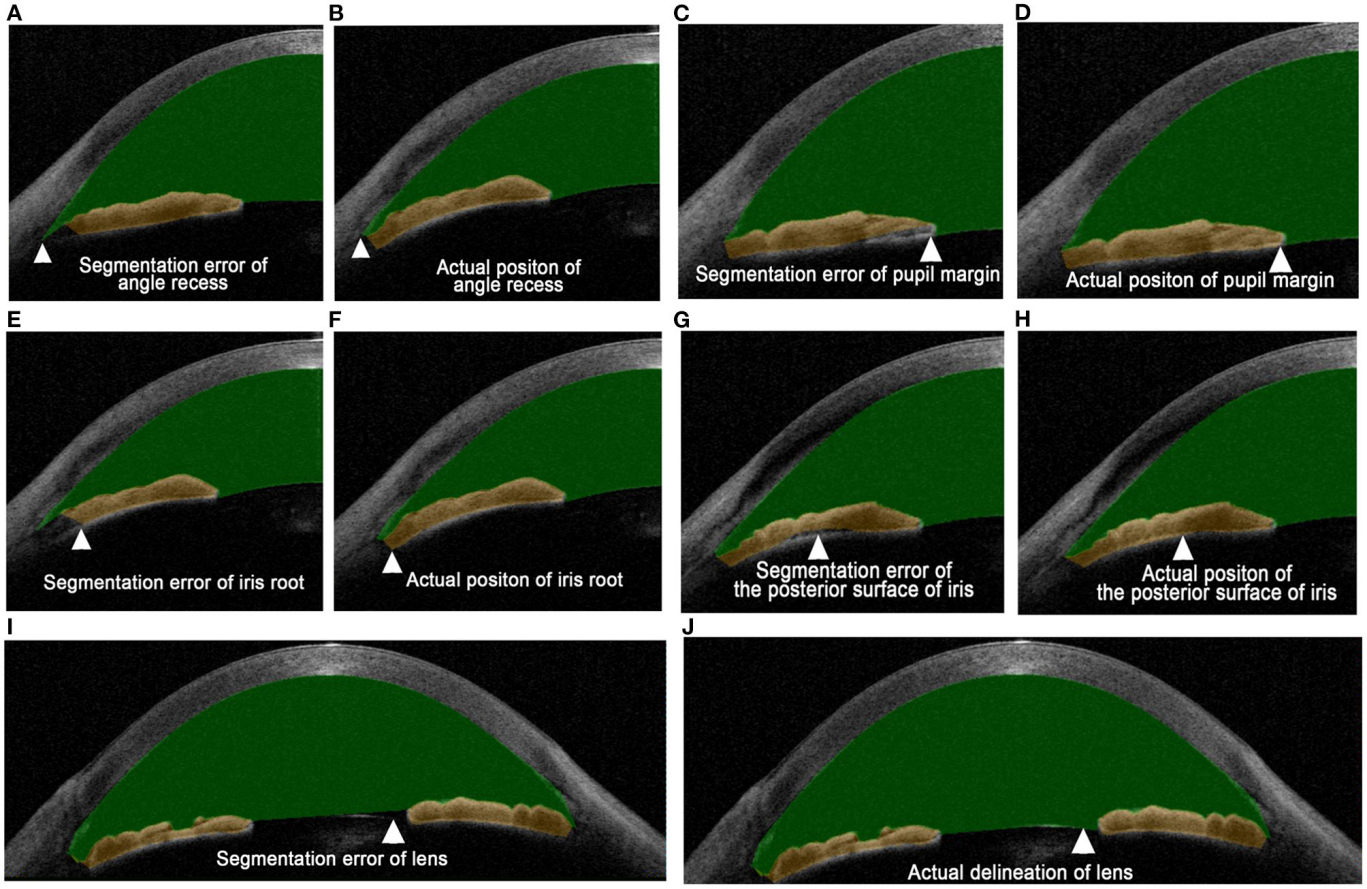
Definitions of different types of segmentation errors. **(A)** Angle recess segmentation error occurs when the posterior boundary of the cornea does not reach the angle recess. **(B)** The angle recess is accurately located after manual correction. **(C)** The pupil margin segmentation error occurs when the boundary of the iris does not conclude the pupil margin. **(D)** The boundary of the iris concludes the pupil margin after manual correction. **(E)** The iris root segmentation error occurs when the boundary of the iris does not conclude the iris root. **(F)** The iris root is concluded in the volume measurement after manual correction. **(G)** The segmentation error of posterior surface of the iris is defined as the dislocation of boundary of the posterior surface of the iris. **(H)** The posterior surface of the iris is accurately delineated after manual correction. **(I)** The segmentation error of lens surface is defined as the dislocation of the boundary of anterior surface of lens. **(J)** The anterior surface of the lens is accurately delineated after manual correction. Arrowheads indicate the location of the segmentation error where the manual correction is carried out.

Comparisons of rates of different segmentation errors between the groups are displayed in Table 2. Segmentation errors were classified by their location. The definition of angle recess segmentation error was that the posterior boundary of the cornea did not reach the angle recess (Figures 2A,B); the definition of a pupil margin segmentation error or iris root segmentation error was that the boundary of iris did not conclude the pupil margin or the iris root (Figures 2C–F); the segmentation error of posterior surface of the iris was defined as the dislocation of boundary of the posterior surface of iris (Figures 2G,H); the segmentation error of lens surface was defined as the dislocation of the boundary of the anterior surface of the lens (Figures 2I,J). A total of 12,800 SS-ASOCT scans were reviewed. Segmentation errors of angle recess, iris root, posterior surface of iris, pupil margin, and anterior surface of lens were noted in 10,760 (84.06%), 11,942 (93.30%), 1,683 (13.15%), 7,579 (59.21%), and 3,234 (25.27%) scans, respectively. Segmentation errors of angle recess, iris root, posterior surface of the iris, and pupil margin occurred more frequently in narrow-angle eyes than wide-angle eyes, while more segmentation errors of the anterior surface of the lens were found in wide-angle eyes (all *P* < 0.001).

**Table 2:** Comparison of segmentation error rates of narrow-angle and wide-angle eyes.

**Categories**	**Total**	**Narrow angle**	**Wide angle**	***P* value**
	**(*n* = 12,800)**	**(*n* = 6,400)**	**(*n* = 6,400)**	
Angle recess	10,760 (84.06%)	5,520 (86.24%)	5,240 (81.88%)	**<0.001**
Iris root	11,942 (93.30%)	6,135 (95.86%)	5,807 (90.73%)	**<0.001**
Posterior surface of iris	1,683 (13.15%)	943 (14.74%)	740 (11.56%)	**<0.001**
Pupil margin	7,579 (59.21%)	4,546 (71.03%)	3,033 (47.40%)	**<0.001**
Anterior surface of lens	3,234 (25.27%)	1,407 (21.98%)	1,827 (28.54%)	**<0.001**

*n refers to numbers of SS-ASOCT scans reviewed. Data are presented as no. (%). Bold values indicate statistical significance*.

Comparisons of volume parameters before and after manual correction of segmentation errors between the two groups are shown in Table 3. ACV decreased and IV increased significantly after manual correction of segmentation errors in the narrow-angle group (*P* = 0.003 and 0.001, respectively) and the wide-angle group (both *P* ≤ 0.001). As for changes in ACV and IV before and after correction (ΔACV, ΔIV), as well as the percentage of ΔACV or ΔIV in actual ACV or IV (ΔACV/ACV-AC and ΔIV/IV-AC), there were no significant differences between narrow-angle and wide-angle eyes (all *P* > 0.05).

**Table 3:** Comparison of volume parameters of two groups before and after manual correction.

	**Narrow-angle**	**Wide-angle**	***P* value**
**ACV (mm**3**)**			
Before correction (ACV-BC)	85.47 ± 19.40	159.34 ± 26.37	
After correction (ACV-AC)	84.46 ± 19.51	157.51 ± 26.71	
ΔACV	−1.00 ± 0.78	−1.83 ± 1.13	0.09
ΔACV / ACV-AC, %	−1.18 ± 1.06	−1.16 ± 0.76	0.89
*P* value (ACV-BC vs. ACV-AC)	**0.003**	**0.001**	
**IV (mm**3**)**			
Before correction (IV-BC)	29.94 ± 5.99	38.38 ± 2.73	
After correction (IV-AC)	33.43 ± 5.56	41.49 ± 2.46	
ΔIV	3.49 ± 2.10	3.11 ± 1.13	0.61
ΔIV / IV-AC, %	10.43 ± 11.23	7.50 ± 3.24	0.450
*P* value (IV-BC vs. IV-AC)	**0.001**	**<0.001**	

*Data are presented as mean ± SD. Bold values indicate statistical significance. ACV, anterior chamber volume; IV, iris volume; and Δ, changes in volume before and after manual correction*.

Associations between total segmentation error rates of different types and anterior segment parameters were evaluated by univariable logistic regression analysis (Table 4). Smaller ACD was significantly associated with more segmentation errors in angle recess, iris root, posterior surface of the iris, and pupil margin, while larger ACD was associated with more incorrect delineation of the anterior surface of lens (all *P* < 0.001). In addition, a thicker CCT was associated with more segmentation errors of the anterior surface of lens (*P* < 0.001). Besides, smaller ACW was associated with more segmentation errors in angle recess (*P* = 0.001) and iris root (*P* < 0.001), while larger ACW was associated with more failure of delineation of the anterior surface of lens (*P* < 0.001). Furthermore, larger LV was associated with more angle recess segmentation error (*P* < 0.001).

**Table 4:** Univariable logistic regression analysis of the total segmentation error rates in different categories.

**Characteristic**	**Angle recess**		**Iris root**		**Posterior surface of iris**		**Pupil margin**		**Anterior surface of lens**	
	**OR, (95% CI)**	***P***	**OR, (95% CI)**	***P***	**OR, (95% CI)**	***P***	**OR, (95% CI)**	***P***	**OR, (95% CI)**	***P***
ACD	0.11 (0.03–0.40)	**<0.001**	0.03 (0.01–0.06)	**<0.001**	0.09 (0.05–0.13)	**<0.001**	0.12 (0.09–0.16)	**<0.001**	4.07 (1.40–6.87)	**<0.001**
CCT	1.03 (0.65–1.75)	0.470	0.66 (0.03–1.19)	0.387	1.30 (0.37–2.69)	0.486	0.76 (0.45–2.23)	0.753	1.02 (1.01–1.03)	**<0.001**
LV	5.30 (2.72–7.37)	**<0.001**	2.40 (0.57–10.03)	0.230	1.69 (0.61–4.71)	0.315	2.42 (0.72–8.16)	0.154	2.85 (0.65–12.55)	0.165
ACW	0.652 (0.51–0.84)	**0.001**	0.016 (0.003–0.09)	**<0.001**	1.50 (0.48–4.69)	0.486	0.96 (0.75–1.23)	0.753	8.32 (3.42–20.25)	**<0.001**

*Bold values indicate statistical significance. OR, odds ratio; CI, confidence interval; ACD, anterior chamber depth; CCT, central corneal thickness; LV, lens vault; ACW, anterior chamber width*.

## Discussion

Swept-source anterior segment OCT provides objective and repeatable measurements of the anterior segment structures (15). Studies have revealed that accurate measurement of ACV and IV played an important role in screening, diagnosis, and treatment decisions for patients with glaucoma, especially for narrow-angle individuals. However, in the current study, we found that failure of correct segmentation algorithms in the measurement of ACV and IV was very common even in images with good quality. Segmentation errors of angle recess, iris root, posterior surface of iris, and pupil margin were more frequent in narrow-angle eyes while the error rate of the anterior surface of lens was higher in wide-angle eyes. SS-ASOCT automated segmentation errors resulted in larger ACV and smaller IV, which were associated with ACD, ACW, LV, and CCT. To the best of our knowledge, this is the first study investigating the error rate of segmentation and its determinants in the automatic measurements of ACV and IV using SS-ASOCT.

The misidentification of iris root was the most common algorithm software–related error, counting 93.3% in this study. The main limitation of SS-ASOCT is that light energy is unable to penetrate tissues behind the iris pigment epithelium. Therefore, the ciliary body cannot be visualized in SS-ASOCT images (2). Thus, the iris root, which inserts into the ciliary body, was probably unidentifiable in most SS-ASOCT images. As shown in our previous study and other studies, ciliary bodies were thinner and more anteriorly rotated in eyes with primary angle closure glaucoma as well as in their fellow eyes (16–18). With the anteriorly rotated ciliary bodies plastering to the posterior surface of the iris, iris root in narrow-angle eyes is harder to be recognized, and therefore the error rate of iris root was higher in narrow-angle eyes. Furthermore, in this study, smaller ACD and ACW were associated with more segmentation errors of iris root, indicating that the recognition of iris root was affected by the degree of stenosis of anterior chamber angle.

In the current study, the segmentation error rate of angle recess was as high as 84.06%. The deviation in the automatic location of scleral spurs by the built-in software of SS-ASOCT leads to segmentation errors of angle recess. In 15–28% of ASOCT images, operators were unable to identify the scleral spur, which made the automatic identification of angle recess difficult (19, 20). To conduct an accurate measurement of volume parameters with SS-ASOCT, manual adjustment of the scleral spur was usually made (3). SS-ASOCT software performs the automated segmentation through the identification of the difference of signal intensity between adjacent layers (21). However, in narrow-angle eyes, the distance from the iris to the scleral spur in the angle is short and the scleral spur is difficult to be detected precisely, leading to more segmentation errors of angle recess in these eyes (22). In the current study, smaller ACD, ACW, and larger LV were associated with more segmentation errors in angle recess, indicating that the shallow anterior chamber and narrow angle added more difficulty in discriminating the angle structures.

The position of the lens is far away from the zero optical path plane of the B-scan, which may result in segmentation errors of the anterior surface of lens. For the same reason, more segmentation errors of the anterior surface of lens were found in wide-angle eyes which had a deeper anterior chamber, and were associated with thicker CCT. On the contrary, narrow-angle eyes had smaller ACD and larger LV, which meant a shorter distance from the lens to the zero optical path plane, making the imaging of the anterior surface of lens clearer and reducing the segmentation error.

The optic signal of ASOCT significantly attenuates when penetrating iris. The contrast effect of the intraocular structure reduces, resulting in more errors in the segmentation algorithm (23, 24). Thus, the boundary of the posterior surface of the iris and pupil margin was misidentified by the built-in software in some scans. In the previous studies, iris curvature is larger in narrow-angle eyes, which indicates that the iris bent more forwardly to the cornea because of a pupillary block (25, 26). In this condition, the iris surface is obviously tilted, and an oblique transmission of OCT light is expected, leading to an improper alignment of the OCT light and image blurring of the OCT scan (10). The same problem was found in OCT imaging of the retina in high myopia eyes and age-related macular degeneration eyes (27, 28). Therefore, segmentation errors in posterior surface of the iris were more frequent in narrow-angle eyes. Otherwise, because of pupillary block in narrow-angle eyes, the margin of iris was plastered to the surface of lens, which makes the pupil margin harder to be recognized. Since ACD could be an indicator of the extent of pupillary block (22), smaller ACD could result in more segmentation errors of the posterior surface of the iris and pupil margin in the current study.

In this study, ACV decreased and IV increased significantly after manual correction of segmentation errors in the two groups (Table 3). According to Figure 2A, the angle recess was always located incorrectly on the ciliary body, which occurred in 84.06% of the B-scans. The retrodeviation of the positioning of angle recess led to an overestimation of the ACV. According to Figures 2I,J, ACV increased after the correction of segmentation errors in the anterior surface of lens. But segmentation errors of the anterior surface of lens only occurred in 25.27% of the B-scans, which had less effect on ACV than segmentation errors of angle recess. On the other hand, according to Figures 2C,E,G, the automated segmentation of the pupil margin, iris root, and posterior surface of the iris did not reach the actual border of the iris, which accounts for 93.30, 13.15, and 59.21%, respectively. These errors made IV significantly smaller than its actual value. The remarkable effect of the correction of these errors indicated that an accurate measurement of the ACV and IV required a manual correction of the segmentation after the automatic measurement or an update of the built-in software of SS-ASOCT to improve the accuracy of image edge recognition.

There were several limitations in this study. First, because the subjects of this study were recruited from the Guangzhou Diabetic Eye Study, eyes with peripheral anterior synechiae in the angle were not included, and the results of the current study are not applicable to angle-closure eyes. However, considering that segmentation errors by SS-ASOCT were more prevalent in narrow-angle eyes in the current research, it is probable that the automated segmentation is more inaccurate and unreliable in angle-closure eyes, in which the angle structures are more difficult to discriminate. Further research should be conducted to analyze the segmentation errors in eyes with iridotrabecular contact. Second, since we did not investigate the segmentation errors in other ASOCT devices or segmentation algorithms, our results are only applicable to CASIA SS-1000 OCT and the current built-in software (V.7J.8, Tomey, Nagoya, Japan).

In conclusion, segmentation errors were prevalent in the automatic measurement of ACV and IV by SS-ASOCT, particularly in narrow-angle eyes, leading to an overestimation of ACV and underestimation of IV. Manual correction of the segmentation errors after the automatic measurement or an update of the built-in software of SS-ASOCT to improve the accuracy of image edge recognition should be considered.

## Data Availability Statement

The raw data supporting the conclusions of this article will be made available by the authors, without undue reservation.

## Ethics Statement

The studies involving human participants were reviewed and approved by Zhongshan Ophthalmic Center, Sun Yat-sen University. The patients/participants provided their written informed consent to participate in this study.

## Author Contributions

All authors listed have made a substantial, direct, and intellectual contribution to the work and approved it for publication.

## Funding

This work was supported by the Natural Science Foundation of Guangdong Province in China (Grant No. 2021A1515012142).

## Conflict of Interest

The authors declare that the research was conducted in the absence of any commercial or financial relationships that could be construed as a potential conflict of interest.

## Publisher's Note

All claims expressed in this article are solely those of the authors and do not necessarily represent those of their affiliated organizations, or those of the publisher, the editors and the reviewers. Any product that may be evaluated in this article, or claim that may be made by its manufacturer, is not guaranteed or endorsed by the publisher.
